# Genetic factors and comorbid pathologies interact to drive regional mitophagy alterations in Lewy body dementia

**DOI:** 10.1007/s00401-025-02964-6

**Published:** 2025-12-01

**Authors:** Xu Hou, Tyrique Richardson, Michael G. Heckman, Fabienne C. Fiesel, Launia J. White, Shunsuke Koga, Owen A. Ross, Dennis W. Dickson, Wolfdieter Springer

**Affiliations:** 1 Department of Neuroscience, Mayo Clinic, Jacksonville, FL USA; 2 Neuroscience PhD Program, Mayo Clinic Graduate School of Biomedical Sciences, Jacksonville, FL USA; 3 Division of Clinical Trials and Biostatistics, Mayo Clinic, Jacksonville, FL USA

**Keywords:** APOE4, α-Synuclein, Mitochondria, Mitophagy, Ubiquitin, PINK1, PARK2, Parkin, Tau, ZMIZ1

## Abstract

The kinase–ligase pair PINK1–PRKN initiates mitophagy by recognizing and selectively tagging worn-out and dysfunctional mitochondria with phosphorylated ubiquitin (pS65-Ub) to facilitate their elimination via autophagy. In human autopsy brains, the number of pS65-Ub positive cells increases with age but is also associated with Lewy body (LB), neurofibrillary tangles (NFT), and senile plaque (SP) burden. Through a recent genome-wide association study, we identified two genetic modifiers of pS65-Ub levels, *APOE4* and *ZMIZ1* rs6480922. While LB, NFT, and SP pathologies often coexist in Lewy body dementia (LBD), it is unclear how genetic factors and comorbid neuropathologies interact to impact mitophagy in vulnerable brain regions. We therefore measured levels of the age and disease marker pS65-Ub in the hippocampus and amygdala of 371 LBD cases. Significant and independent associations with pS65-Ub levels were observed for each of the three pathologies LB, NFT, and SP in both regions, and the presence of *APOE4* significantly strengthened the association between NFT and pS65-Ub in the hippocampus. While no interaction between LB and SP pathologies was observed regarding association with pS65-Ub, a significant interaction between LB and NFT pathologies on pS65-Ub accumulation was found in the amygdala, which was primarily observed in carriers of the minor allele of *ZMIZ1* rs6480922. In summary, our study revealed complex interactions between LB pathology, NFT pathology, and genetic mitophagy modifiers in LBD brains, highlighting potential convergent molecular mechanisms underlying α-synuclein- and tau-associated mitophagy alterations.

**Supplementary Information:**

The online version contains supplementary material available at 10.1007/s00401-025-02964-6.

## Introduction

Lewy body dementia (LBD) is a complex neurodegenerative disorder and the second most common cause of dementia in the elderly after Alzheimer’s disease (AD). LBD lies on the spectrum between Parkinson’s disease (PD) and AD, yet maintains distinct clinical and pathological features. Patients with LBD typically present with progressive dementia and often experience a combination of cognitive decline, spontaneous parkinsonism, visual hallucinations, and REM sleep behavior disorder [1]. Neuropathologically, LBD is characterized by the prevalent accumulation of misfolded α-synuclein in the form of Lewy bodies (LBs) and Lewy neurites [2], which extend beyond the brainstem into the limbic system and various cortical regions [3, 4]. In contrast to AD which is defined by a strong deposition of tau neurofibrillary tangles (NFTs) and amyloid β-containing senile plaques (SPs), LBD cases typically present with a mild to moderate burden of AD-type pathology, particularly for tangle-related changes [3, 5, 6]. This convergence of multiple proteinopathies contributes to the clinical and pathological heterogeneity of LBD and complicates the diagnosis, treatment, and understanding of disease mechanisms. Recent genetic studies have identified several risk loci associated with LBD, including variants in *APOE*, *GBA*, and *SNCA* [7–11]**.** These findings suggest that LBD shares overlapping genetic risk factors with both PD and AD, while also presenting a unique genetic architecture that may influence disease onset, progression, and therapeutic response.

Mitochondrial dysfunction is increasingly recognized as a shared and early feature across aging and neurodegeneration, where it may contribute to the misfolding and aggregation of pathological proteins such as α-synuclein, tau, and amyloid β [12–14]. To preserve mitochondrial integrity and cellular homeostasis, cells employ a selective form of autophagy, named mitophagy, to target damaged mitochondria for lysosomal degradation [15]. The best-characterized mitophagy pathway is directed by the ubiquitin kinase–ligase pair PINK1–PRKN [16, 17]. In this pathway, PINK1 accumulates on the outer mitochondrial membrane when its protein import is blocked and subsequently recruits PRKN, which catalyzes the ubiquitination of mitochondrial surface proteins. A critical step in this cascade is the phosphorylation of ubiquitin at serine 65 (pS65-Ub) by PINK1, which serves as a molecular tag on damaged mitochondria to facilitate the recruitment of autophagic receptors and promote their subsequent lysosomal clearance [18]. Under physiological conditions, PINK1–PRKN signaling ensures the efficient turnover of pS65-Ub tagged dysfunctional mitochondria, thereby maintaining relatively low baseline levels of pS65-Ub. Although typically transient, pS65-Ub levels markedly increase with normal aging in mice and in human brain [18–20], which may result from increased mitochondrial damage and activation of PINK1–PRKN and/or from impaired degradation and inefficient flux through lysosomes. Changes in mitophagy flux during aging have recently been shown to differ across brain regions and even among specific cell types in mice [21], suggesting the presence of complex and diverse regulatory mechanisms.

Independently of the normal aging contribution, pS65-Ub levels are even further elevated in a variety of neurodegenerative diseases, including AD, PD, and frontotemporal dementia with parkinsonism [20, 22–26]. Across this spectrum of diseases the accumulation of pS65-Ub significantly associates with α-synuclein and tau pathology [20, 23]. Given the frequent co-occurrence of LB, SP and NFT pathologies in LBD, we here aimed to assess their independent association with the mitophagy marker pS65-Ub and analyze how the core α-synuclein pathology interacts with comorbid AD-type pathology (tau or amyloid β) to impact mitophagy alteration using a cohort of 371 autopsy-defined LBD cases. We recently employed pS65-Ub levels as a quantitative disease endophenotype and through a genome-wide association study identified two genetic modifiers with opposing effects [27]. As a major disease risk factor, *APOE* rs429358 (*APOE4*) is strongly associated with higher pS65-Ub levels and greater pathological burden, while *ZMIZ1* rs6480922 is associated with decreased pS65-Ub, greater brain weight, and reduced hippocampal neuropathologies, suggesting a potential resilience role. Here, we further assessed interactions between the two genetic mitophagy modifiers and the comorbid pathologies in relation to pS65-Ub levels in our LBD series.

We focused our analysis on the hippocampus and the amygdala due to their critical roles in memory and emotional regulation as well as their central involvement in the clinical manifestation of LBD including cognitive fluctuations, memory impairment, and neuropsychiatric symptoms [1, 28–31]. More importantly, these two regions are among the early and most consistently affected regions in LBD, harboring all three types of neuropathologies including LBs SPs, and NFTs [4, 32]. In a pathologically defined large autopsy LBD cohort, our results indicate complex regional interactions between genetics, biology, and pathology. While each of the three main pathologies significantly and independently contributes to pS65-Ub alterations regardless of brain region, our findings further highlight a synergistic interaction between LB and NFT pathology specifically within the amygdala that may be partially mediated by *ZMIZ1* rs6480922. In the future, it will be critical to define the biological and/or pathological mechanisms underlying the abnormal buildup of pS65-Ub signals in disease brains and to identify therapeutic targets that can restore mitophagy flux and attenuate disease progression.

## Materials and methods

### Study design and subjects

A total of 371 LBD cases were obtained from the Mayo Clinic Florida Brain Bank for neurodegenerative disorders. This cohort was systematically screened for known pathogenic mutations for α-synucleinopathy with only one case found to carry a duplication in the *SNCA* gene. This case was nevertheless included in all analyses as its inclusion or exclusion did not affect the results. All of these autopsy-defined LBD cases were selected to have a high likelihood of clinical LBD syndrome [3] (Table 1). More specifically, all cases exhibited either (a) transitional or diffuse LB pathology combined with Braak tangle stages 0 to II, or (b) diffuse LB pathology combined with Braak tangle stages III to IV [3]. Neuropathological assessments were conducted in a systematic and standardized manner by a single neuropathologist as previously described [33]. Demographic information (age at death, sex, and race) and neuropathological characteristics from the hippocampus and amygdala were collected for all cases. Neuropathological assessments included measures of α-synuclein pathology (Lewy body disease subtype [transitional or diffuse] and LB density, by NACP immunohistochemical staining pretreated with formic acid), amyloid pathology (Thal amyloid phase and SP density, by thioflavin S), and tau pathology (Braak tangle stage and NFT density, by thioflavin S). Manual quantification of LB, SP, and NFT densities was performed as counts per microscopic field with a maximum cap of 50 counts.

**Table 1:** Characteristics of Lewy body dementia cohort

Variable	*N*	Median (minimum, maximum) or No. (%) of subjects
Age at death (years)	371	77 (54, 94)
Sex (Male)	371	250 (67.4%)
Race (Caucasian)	371	365 (98.4%)
α-Synuclein pathology measures		
Lewy body disease subtype	371	
Transitional Lewy body disease		71 (19.1%)
Diffuse Lewy body disease		300 (80.9%)
Hippocampal LB density (/microscopic field)	278	0.9 (0.0, 6.1)
Amygdala LB density (/microscopic field)	366	50 (4, 50)
Amyloid pathology measures		
Thal amyloid phase	371	
0		45 (12.1%)
1		34 (9.2%)
2		35 (9.4%)
3		132 (35.6%)
4		47 (12.7%)
5		78 (21.0%)
Hippocampal SP density (/microscopic field)	275	6 (0, 50)
Amygdala SP density (/microscopic field)	367	18 (0, 50)
Tau pathology measures		
Braak tangle stage	371	
0		12 (3.2%)
I		18 (4.9%)
II		106 (28.6%)
III		85 (22.9%)
IV		150 (40.4%)
V		0 (0.0%)
VI		0 (0.0%)
Hippocampal NFT density (/microscopic field)	275	2 (0, 40)
Amygdala NFT density (/microscopic field)	367	1 (0, 50)
pS65-Ub measures		
Hippocampal pS65-Ub density (/mm^2^)	275	1.5 (0.1, 27.3)
Amygdala pS65-Ub density (/mm^2^)	371	0.2 (0.0, 7.2)
Number of *APOE4* alleles	352	
0		186 (52.8%)
1		142 (40.3%)
2		24 (6.8%)
Number of minor alleles of *ZMIZ1* rs6480922	354	
0		206 (58.2%)
1		129 (36.4%)
2		19 (5.4%)

*LB *Lewy body, *SP* senile plaque, *NFT* neurofibrillary tangle

### Immunohistochemistry and image analysis for pS65-Ub

Using an established, mostly automated workflow, immunohistochemical staining of paraffin-embedded postmortem brain tissue containing hippocampus or amygdala was performed as previously described [20, 22, 23]. The paraffin-embedded brain tissue was cut into 5 µm sections and allowed to dry overnight at 60 °C. Slides were deparaffinized and rehydrated, followed by antigen retrieval in steaming deionized water for 30 min. To minimize variability, immunohistochemical staining was performed on the Lab Vision Autostainer 480S (Thermo Fisher Scientific, Waltham, MA, USA) with tightly controlled temperature, humidity, and timing throughout the procedure and between runs. Specifically, after blocking with 0.03% hydrogen peroxide and 5% normal goat serum (Invitrogen, 16,210,072), sections were incubated with primary antibody against pS65-Ub (in-house [18], 1:650), followed by rabbit-labeled polymer HRP (Agilent, K4011) at room temperature. Peroxidase labeling was visualized with the chromogen solution 3,3’-diaminobenzidine (Agilent, K346811-2) before sections were counterstained. Sections were sequentially dehydrated and coverslipped in a Leica Autostainer XL and a Leica glass coverslipper CV 5030 (Leica Biosystems, Wetzlar, Germany), respectively. After staining, all sections were scanned with an Aperio AT2 digital pathology scanner (Leica Biosystems, Wetzlar, Germany) and then traced and quantified using optimized Aperio algorithms to count the positive cell number followed by manual quality control.

### Genotyping for *APOE4* and *ZMIZ1* rs6480922

Frozen cerebellum brain tissue was collected from all cases and genomic DNA was extracted using Autogen Flex Star methods. As previously described [27], samples were genotyped using a custom Taqman SNP genotyping assay for *APOE4* (i.e., *APOE* rs429358) and a custom MassARRAY® System iPlex assay for *ZMIZ1* rs6480922. There was no evidence of a departure from Hardy–Weinberg equilibrium for either variant (*p* ≥ 0.79).

### Groupwise and multivariable linear regression analysis

Continuous variables were summarized with the sample median and range. Categorical variables were summarized with number and percentage. Comparisons of pS65-Ub, LB density, SP density, and NFT density between the hippocampus and the amygdala were made using a paired Wilcoxon signed rank test. To evaluate whether independent associations exist between pS65-Ub and each pathological measure (LB, SP, or NFT density) in the hippocampus and amygdala, we used multivariable linear regression models where all measures except amygdala LB and SP density were assessed on the cube root scale due to the presence of skewed distributions. These values were subsequently scaled to have mean = 0 and standard deviation = 1 in order to enhance the interpretability of results of regression analyses. β coefficients and 95% confidence intervals were estimated and are interpreted as the change in mean pS65-Ub level (on the cube root scale and after subsequent scaling) corresponding to each 1-standard deviation increase (after cube root transformation when applicable) in LB, SP, or NFT density. The multivariable linear regression models were adjusted for age at death and sex, and also for the two other pathological measures (e.g., SP and NFT densities were adjusted for when examining associations of pS65-Ub with LB density). We considered *p*-values ≤ 0.0167 as statistically significant after applying a Bonferroni correction for multiple testing for the three pathological measures that were assessed for association with pS65-Ub level in a given brain region. Of note, in this and all subsequently described regression analyses, these were performed on a brain region-specific basis; amygdala measures were only assessed for association with other amygdala measures, and hippocampus measures were only examined for association with other hippocampus measures.

### Two-way and three-way interaction analysis

We next assessed whether the aforementioned associations were consistent in carriers and non-carriers of *APOE4*, and carriers and non-carriers of the minor allele of *ZMIZ1* rs6480922, by adding an interaction term between LB, SP, or NFT measures and the presence of *APOE4* or the presence of the minor allele of *ZMIZ1* rs6480922 into the aforementioned multivariable regression models (individual covariates for *APOE4* or *ZMIZ1* rs6480922 were also included in the models). *P*-values ≤ 0.0167 were considered statistically significant in this portion of the analysis after applying a Bonferroni correction for multiple testing for the three tests of associations or interactions that were performed in each separate brain region.

Interactions of LB pathology with SP and NFT pathology in regard to association with pS65-Ub levels in a given brain region were assessed using multivariable linear regression models that included covariates for age at death, sex, LB density, SP density, NFT density, as well as the interaction of LB density with the given pathological measure of interest (SP or NFT density). *P*-values ≤ 0.025 were considered statistically significant after applying a Bonferroni correction for multiple testing for the two different tests of interactions that were performed in a given brain region. Finally, we evaluated whether a detected interaction between LB density and NFT density was consistent between carriers and non-carriers of *APOE4*, or between carriers and non-carriers of the minor allele of *ZMIZ1* rs6480922 by adding an interaction with *APOE4* or *ZMIZ1* rs6480922 into the aforementioned interaction terms (i.e., a three-way interaction). Other covariates included age at death, sex, LB density, SP density, NFT density, and all three pairwise interactions for the three variables that were included in the three-way interaction. *P*-values ≤ 0.025 were considered statistically significant after making a Bonferroni correction for the two different three-way interactions that were assessed. All statistical tests were two-sided and were performed using SAS (version 9.4; SAS Institute, Inc., Cary, North Carolina).

## Results

### Distinct regional distribution and independent associations of pS65-Ub with neuropathological burden in LBD

**Fig. 1:**
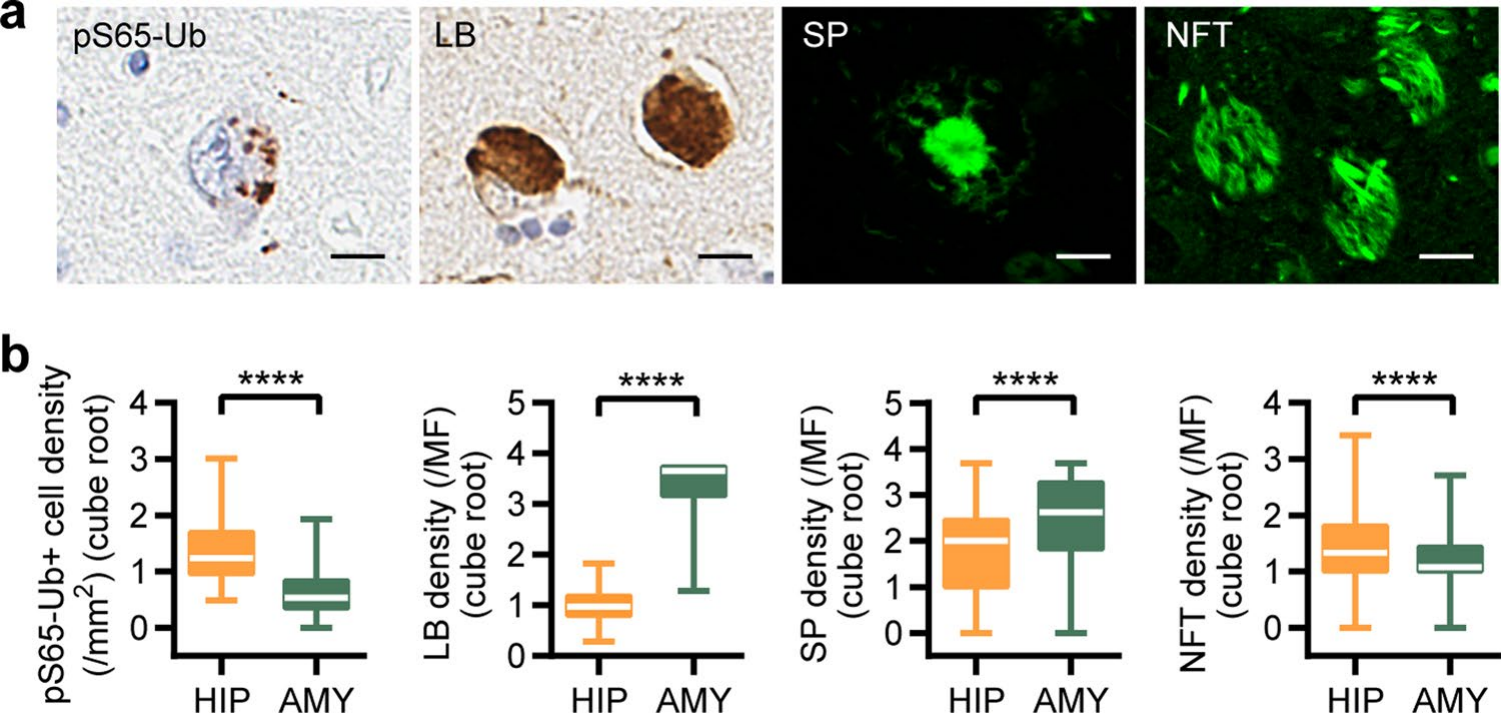
Differential distribution of pS65-Ub inclusions and neuropathological deposits in the hippocampus and amygdala of LBD. (**a**) Representative images of pS65-Ub positive cells, LBs, SPs, and NFTs in LBD brain. Scale bar: 10 µm. (**b**) Hippocampus shows significantly higher pS65-Ub positive cell density and NFT deposition, while the amygdala contains greater LB and SP pathology compared to the hippocampus. Data are shown on a cube root scale. Box-and-whisker plots extend from the minimum to the maximum values. Paired Wilcoxon signed rank test, ***p < 0.0001. LB—Lewy body, SP—senile plaque, NFT—neurofibrillary tangles, HIP—hippocampus, AMY—amygdala, MF—microscopic field

We first assessed pS65-Ub levels and burdens of LB, SP, and NFT in the hippocampus and amygdala of 371 LBD cases. Consistent with previous findings [20, 23, 24], we observed a marked accumulation of pS65-Ub-labeled granular structures in both regions (Fig. 1a). Unbiased quantification revealed significantly higher levels of both NFT density and especially pS65-Ub positive cell density in the hippocampus (both *p* < 0.0001, Fig. 1b). In contrast, the amygdala showed pronounced accumulation of LBs and a significant increase in SP density (both *p* < 0.0001, Fig. 1b). To characterize the impact of key neuropathological features on mitophagy alterations, we next examined whether independent associations exist between pS65-Ub levels and densities of LBs, SPs, and NFTs in both brain regions (Table 2). Multivariable linear regression models that were adjusted for age at death, sex, LB density, SP density, and NFT density revealed independent and significant associations between pS65-Ub and each neuropathology measure in both the hippocampus (LB: β: 0.15, *p* = 0.001; SP: β: 0.22, *p* = 0.0001; NFT: β: 0.50, *p* = 2.35 × 10^–15^) and the amygdala (LB: β: 0.16, *p* = 0.0005; SP: β: 0.31, *p* = 5.53 × 10^–9^; NFT: β: 0.31, *p* = 9.32 × 10^–9^) (Table 2). Associations with pS65-Ub were stronger for NFT and SP burden than for LB burden, and the association between pS65-Ub and NFT levels was particularly strong in the hippocampus (Table 2). Together, these findings demonstrate that pS65-Ub levels are independently and differentially linked to comorbid neuropathologies in the hippocampus and amygdala.

**Table 2:** Associations of pS65-Ub with neuropathology in Lewy body dementia

Association with pS65-Ub when adjusting for age at death, sex, and other neuropathology^1^	Hippocampus		Amygdala	
	β (95% CI)	*p* value	β (95% CI)	*p* value
LB density	0.15 (0.06, 0.24)	**0.001**	0.16 (0.07, 0.25)	**0.0005**
SP density	0.22 (0.11, 0.32)	**0.0001**	0.31 (0.21, 0.41)	**5.53 × 10**^**–9**^
NFT density	0.50 (0.38, 0.62)	**2.35 × 10**^**–15**^	0.31 (0.21, 0.41)	**9.32 × 10**^**–9**^

*β* regression coefficient, *CI* confidence interval, *LB* Lewy body, *SP* senile plaque, *NFT* neurofibrillary tangle

β values, 95% CIs, and *p* values result from linear regression models

β coefficients are interpreted as the increase in mean pS65-Ub level (on the cube root scale and after subsequent scaling to mean = 0 and standard deviation = 1) corresponding to each 1-standard deviation increase (after cube root transformation when applicable) of LB density, SP density, or NFT density

^1^Adjustment for other neuropathology was done as follows. Models directly involving LB density were adjusted for SP and NFT density; models directly involving SP density were adjusted for LB and NFT density; models directly involving NFT density were adjusted for LB and SP density. *p* values ≤ 0.0167 were considered as statistically significant after applying Bonferroni correction for multiple testing for the three neuropathological measures (LB, SP, and NFT density) that were examined for association with pS65-Ub level in a given brain region. Significant *p* values are shown in bold

### *APOE4* exacerbates tau tangle-associated mitophagy alterations in LBD

**Fig. 2:**
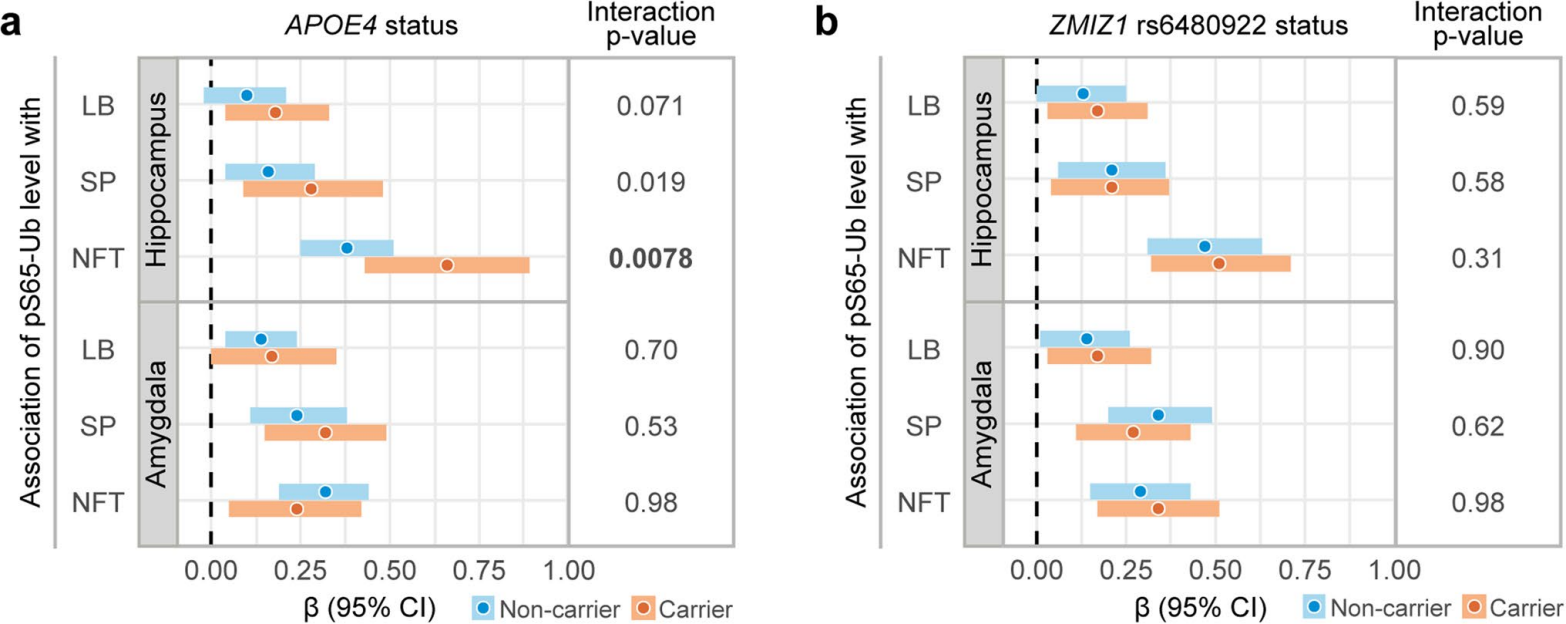
Interactions between genetic factors and individual neuropathology on pS65-Ub accumulation in LBD. Forest plots show the associations between pS65-Ub positive cell density and individual neuropathological burdens (LB, SP, and NFT) in the hippocampus (top) and amygdala (bottom), stratified by either *APOE4* (**a**) or *ZMIZ1* rs6480922 minor allele (**b**) status. Blue bars represent non-carriers and orange bars represent carriers. Filled circles denote β coefficient and horizontal bars indicate 95% confidence intervals. *P*-values indicate the interaction between the variable of interest and genetic status. Significant interactions (*p* ≤ 0.0167) were considered statistically significant after applying a Bonferroni correction for multiple testing and are shown in bold. LB—Lewy body, SP—senile plaque, NFT—neurofibrillary tangles, CI—confidence intervals

Building on the findings from our recent genome-wide association study [27], we subsequently assessed whether the aforementioned associations between LB, SP, and NFT pathology with pS65-Ub are consistent according to the presence of genetic mitophagy modifiers *APOE4* and the minor allele of *ZMIZ1* rs6480922. After correcting for multiple testing (*p*-values ≤ 0.0167 considered as significant), multivariable linear regression models revealed a significant interaction between *APOE4* and NFT density regarding the association with pS65-Ub level in the hippocampus (interaction *p* = 0.0078); though highly significant in both groups, the magnitude of this association was much stronger in carriers of *APOE4* (β: 0.66, *p* = 1.10 × 10^–7^) than in non-carriers (β: 0.38, *p* = 1.16 × 10^–7^) (Fig. 2a, Supplementary Table 1). No significant interactions with *APOE4* were observed for LB (interaction *p* = 0.071) or SP density (interaction *p* = 0.019) in the hippocampus, nor for any of the three pathologies in the amygdala (Fig. 2a, Supplementary Table 1). Additionally, no significant interactions were noted between neuropathology measures and the minor allele of *ZMIZ1* rs6480922 in relation to association with pS65-Ub levels (all interaction *p* ≥ 0.31, Fig. 2b Supplementary Table 2). Our findings suggest that *APOE4* amplifies the pathological impact of tau pathology-mediated mitophagy dysfunction specifically in the hippocampus, highlighting potential *APOE4*-mediated regional vulnerabilities in LBD.

### Synergistic effect of α-synuclein and tau pathologies on mitophagy alterations in the amygdala of LBD

**Fig. 3:**
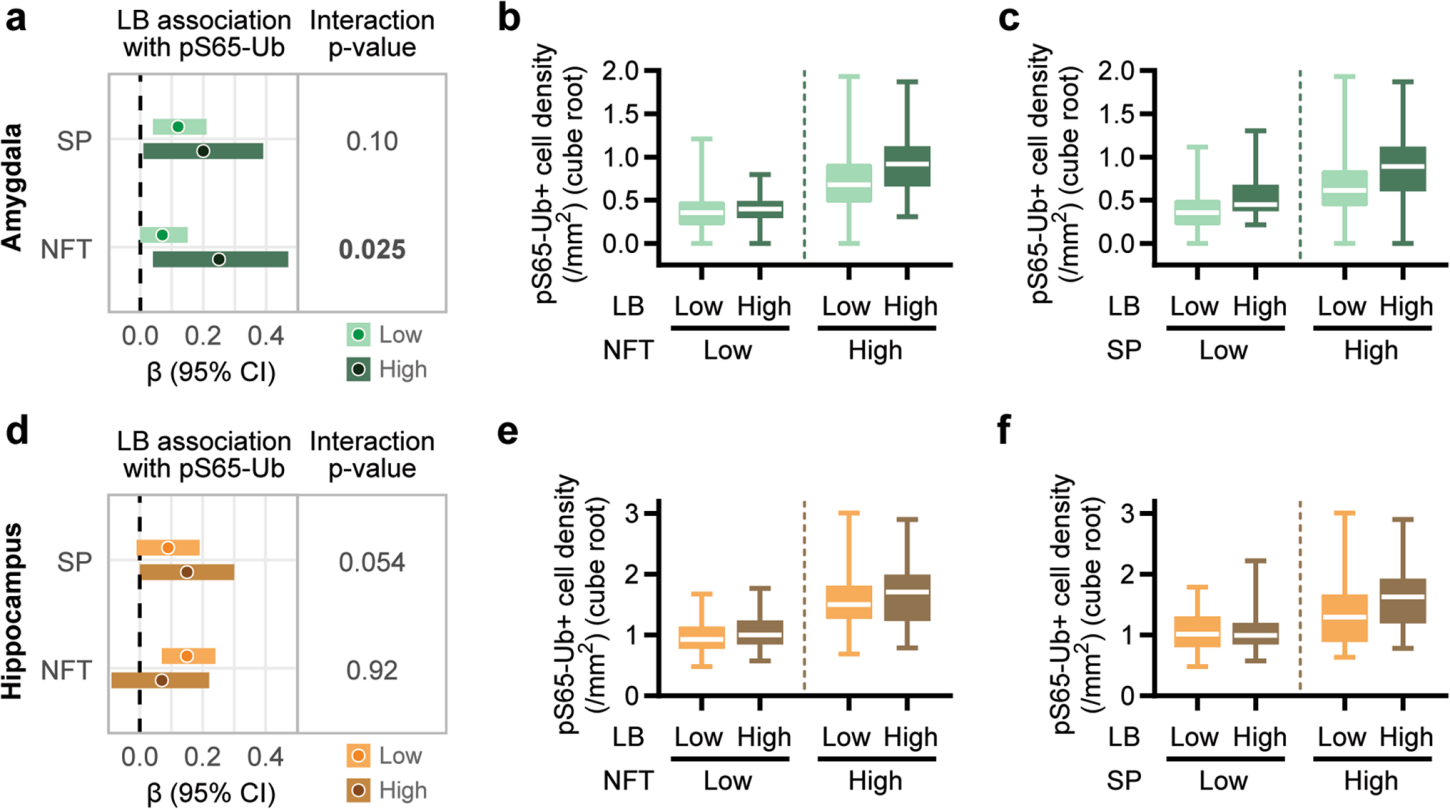
Interaction of comorbid neuropathologies on pS65-Ub accumulation in LBD. (**a, d**) Forest plots show the associations between LB and pS65-Ub levels in subgroups with low or high SP or NFT burden in the amygdala (**a**) and hippocampus (**d**). Bars in lighter color represent low pathology groups and bars in darker color represent high pathology groups. Filled circles denote β coefficient and horizontal bars indicate 95% confidence intervals. *P*-values indicate the interactions between LB and SP or LB and NFT on pS65-Ub levels. Significant interactions (*p* ≤ 0.025) were considered statistically significant after applying a Bonferroni correction for multiple testing and are shown in bold. (**b-c**, **e–f**) Comparison of pS65-Ub positive cell density in LBD cases containing low or high levels of LB, NFT, and SP pathology in the amygdala (**b**-**c**) and hippocampus (**e**–**f**). Data are shown on a cube root scale. Box-and-whisker plots extend from the minimum to the maximum values. LB—Lewy body, NFT—neurofibrillary tangles, SP—senile plaque, CI—confidence intervals

We next investigated potential synergistic effects between the core LB pathology and comorbid SP/NFT pathology on pS65-Ub levels in the hippocampus and amygdala. In these interaction analyses, we identified a significant interaction between LB and NFT density in the amygdala (interaction *p* = 0.025), where the association between LB density and pS65-Ub levels was much stronger for LBD cases with a higher NFT density (β: 0.25, *p* = 0.021) than in cases with a lower NFT density (β: 0.07, *p* = 0.063) (Fig. 3a and b, Supplementary Fig. 1a, Supplementary Table 3). Similar though non-significant trends were observed regarding the interaction between LB and SP density in the amygdala (interaction *p* = 0.10, Fig. 3a and c, Supplementary Fig. 1a, Supplementary Table 3) and the interaction between LB and SP density in the hippocampus (interaction *p* = 0.054, Fig. 3d and f, Supplementary Fig. 1b, Supplementary Table 3). There was no evidence of an interaction between LB density and NFT density regarding association with pS65-Ub in the hippocampus (interaction *p* = 0.92, Fig. 3d and e, Supplementary Fig. 1b, Supplementary Table 3). Collectively, the results suggest that the convergence of α-synuclein and tau pathologies in the amygdala may play a particularly significant role in driving pS65-Ub accumulation in LBD.

### *ZMIZ1* rs6480922 modulates α-synuclein-tau synergy in pS65-Ub accumulation in the amygdala of LBD

**Fig. 4:**
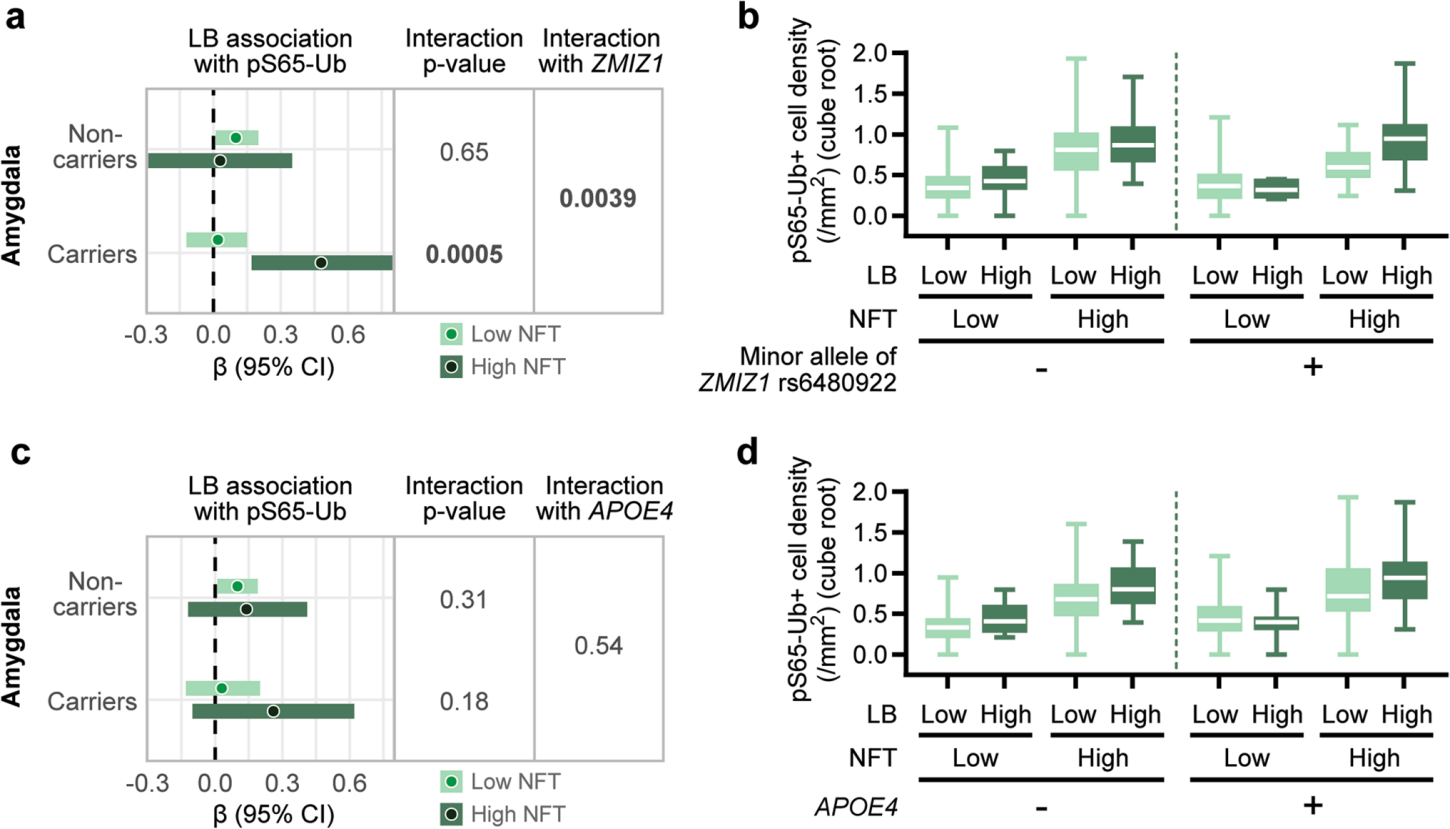
Interactions between genetic factors and comorbid neuropathologies on pS65-Ub accumulation in the amygdala of LBD. (**a, c**) Forest plot illustrates the association between LB and pS65-Ub levels in the amygdala, stratified by NFT levels within non-carriers and carriers of the minor allele of *ZMIZ1* rs6480922 (**a**) or *APOE4* allele (**c**). Bars in lighter color represent low pathology groups and bars in darker color represent high pathology groups. Filled circles denote β coefficient and horizontal bars indicate 95% confidence intervals. *P*-values indicate the interactions between LB and NFT on pS65-Ub levels and additional interaction stratified by *ZMIZ1* rs6480922 or *APOE4* status. Significant interactions (*p* ≤ 0.05) are shown in bold. (**b**, **d**) Comparison of pS65-Ub levels between non-carriers and carriers of the minor allele of *ZMIZ1* rs6480922 (b) or *APOE4* allele (d) in subgroups with low or high levels of LB and NFT pathology in the amygdala. Data are shown on a cube root scale. Box-and-whisker plots extend from the minimum to the maximum values. LB—Lewy body, NFT—neurofibrillary tangles, CI—confidence intervals

To determine whether genetic background drives the observed synergism between α-synuclein and tau pathologies with respect to pS65-Ub levels in the amygdala, we evaluated whether the aforementioned significant interaction between LB and NFT density is consistent between carriers and non-carriers of *APOE4* or the minor allele of *ZMIZ1* rs6480922. In multivariable regression analysis, we identified a significant three-way interaction involving *ZMIZ1* rs6480922 (interaction *p* = 0.0039), where the aforementioned stronger association between LB density and pS65-Ub for LBD cases with a higher NFT density was noted in cases where the minor allele of *ZMIZ1* rs6480922 was present (High NFT: β: 0.48, *p* = 0.003; Low NFT: β: 0.02, *p* = 0.81), but not in cases where the minor allele was absent (High NFT: β: 0.03, *p* = 0.85; Low NFT: β: 0.10, *p* = 0.039) (Fig. 4a and b, Supplementary Table 4). The previously mentioned interaction between LB and NFT density did not differ strongly according to the presence or absence of *APOE4* (interaction *p* = 0.54, Fig. 4c and d, Supplementary Table 4). The results suggest the *ZMIZ1* rs6480922 variant as a potential key modifier for the synergistic interaction between LB and NFT pathologies in driving mitophagy alteration.

## Discussion

In this study, using an autopsy-defined LBD cohort and pS65-Ub as an aging and disease marker, we revealed brain region-specific mitophagy alteration and distinct neuropathological profiles in the hippocampus and amygdala of LBD. We demonstrated strong, independent, yet differential associations between pS65-Ub positive cell density and α-synuclein, amyloid, and tau pathology in these regions. Multivariable linear regression models indicated that *APOE4* significantly amplifies the pathological impact of tau pathology-mediated mitophagy dysfunction specifically in the hippocampus, suggesting a new molecular mechanism by which *APOE4* contributes to the pathogenesis in LBD and AD. Furthermore, using two- and three-way interaction analysis we identified a region-specific synergistic interaction between α-synuclein and tau pathology, but not with amyloid pathology, in driving pS65-Ub levels in the amygdala, and nominated *ZMIZ1* rs6480922 as a potential genetic driver in this pathological interplay. Our study defines regional- and pathological-specific synergism among genetics, biology, and pathology in LBD brains. While contributing to the growing recognition of mixed pathologies in neurodegenerative diseases, these findings underscore mitophagy as a potential biological convergent underlying disease pathogenesis.

pS65-Ub is produced in response to mitochondrial damage through the joint activity of PINK1 and PRKN. While the pathway remains active, pS65-Ub levels stay very low and transient under physiological conditions [34], reflecting the effective “piecemeal” elimination of mitochondrial subdomains that undergo normal wear and tear. The same mechanisms apply, just to a greater extent, upon stress-induced mitochondrial damage such as acute depolarization in cell culture or cumulative cellular stress during aging. This leads to an increased generation of pS65-Ub that may then remain elevated with sustained mitochondrial damage and/or compromised autophagic-lysosomal flux of labeled mitochondria.

This accumulation of pS65-Ub could be further exacerbated in neurodegenerative conditions such as AD, PD, and LBD with the presence of additional pathological, non-degradable substrates. Despite LBs being reportedly absent in some PINK1 or PRKN mutant PD cases, the high abundance of pS65-Ub in non-mutation carriers suggests that the PINK1–PRKN pathway remains active in LBD and even has a complex, disease-context-dependent interaction with LB pathology. While the precise mechanisms underlying the formation and function of these structures remain unclear, recent studies have found enriched mitochondrial and autophagic membrane remnants in LBs in addition to α-synuclein [35, 36], pointing to a potential link with failed mitophagy that warrants further investigation. It is also worth mentioning that although pS65-Ub typically appears as smaller granular structures, it tends to accumulate within larger granulovacuolar degeneration bodies when pre-tangles pathology is present in the same cell [27]. These structures are thought to be late-stage autophagic remnants of incomplete degradation though their functional significance is not yet fully understood [37–39]. As such the mechanisms impairing mitophagy may differ between cells positive for α-synuclein pathology, tau pathology, or both.

We found in LBD that pS65-Ub levels were further elevated in the hippocampus compared to the amygdala. This selective vulnerability of the hippocampus may reflect its heightened bioenergetic demands to sustain synaptic remodeling and adult neurogenesis [40–42], processes that rely heavily on mitochondrial integrity and are particularly susceptible to age- and disease-related dysfunction [41, 43, 44]. Alternatively, this elevation may result from a greater tau burden in the hippocampus given the stronger association between pS65-Ub levels and NFT density than with LB or SP density. Emerging evidence consistently supports a close interplay between tau pathology and mitophagy dysfunction in neurodegenerative diseases [23, 45–49]. Besides the common link to granulovacuolar degeneration bodies, disease-associated tau has been shown to disrupt mitophagy by interfering with PRKN functions in cell and animal models [23, 45, 47, 48]. PRKN overexpression was able to enhance mitophagy flux and reduce tau pathological changes [48].

The *APOE4* allele is a major common genetic risk factor for LBD [7, 8, 11] and has recently been identified as a strong mitophagy modifier that is associated with increased levels of pS65-Ub [27]. The *APOE4* genotype is also well known to result in enhanced tau hyperphosphorylation, aggregation, spreading, and cytotoxicity [50]. Our interaction analysis revealed that the *APOE4* genotype specifically exacerbated the impact of tau pathology-mediated pS65-Ub accumulation in the hippocampus of LBD. However, as a primary lipid transporter in the brain, APOE broadly regulates lipid homeostasis in an isoform-dependent manner across various cell types [51]. Disease-associated *APOE4* promotes lipid accumulation, glia reactivity, oxidative stress, and metabolic shift [51–55], and has been shown to enhance pS65-Ub levels even in model systems without neuropathology [27]. These combined biological and pathological effects of *APOE4* likely converge to exacerbate mitochondrial and lysosomal dysfunction in the pathological environment and might thus promote additional pS65-Ub accumulation in the hippocampus.

We observed a significant synergy of α-synuclein and tau with regard to pS65-Ub accumulation specifically in the amygdala. Besides a strong presence of LB pathology in this region, it may partly be due to the more prominent co-occurrence of both neuropathologies in the same neurons in the amygdala [56, 57]. Pathologic α-synuclein and tau are also known to induce mitochondrial dysfunction and oxidative stress, with tau aggregates amplifying the neurotoxicity on mitochondria by interacting with α-synuclein [58, 59]. This may occur through a convergent action of these two proteins [60] on altering Drp1 localization and disrupting axonal trafficking, both of which are essential for maintaining mitochondrial homeostasis [61, 62]. Additionally, α-synuclein and tau have been reported to interact with autophagic-related proteins such as LC3 and p62/SQSTM1 [63, 64]. Both increased mitochondrial damage and impaired autophagic function may thus underlie the observed synergistic effect of LB and NFT pathology on increasing pS65-Ub in the amygdala.

Our three-way interaction analysis suggests that the *ZMIZ1* rs6480922 variant may be a key driver for the observed synergy. This variant was previously found to be significantly associated with reduced pS65-Ub levels in our genome-wide association study [27]. Interestingly, carriers of the minor allele of rs6480922 in the current study again showed lower pS65-Ub levels, but only in subgroups with high NFT or high LB alone, not in the subgroup with high levels of both pathologies (Fig. 4b). *ZMIZ1* encodes a transcriptional coactivator that enhances transcription mediated by the androgen receptor, p53, SMAD3, and NOTCH signaling [65]. Pathogenic mutations in *ZMIZ1* have been linked to syndromic neural disorders with intellectual disability and neurodevelopmental delay [65]. Of note, knockout of ZMIZ1 in zebrafish resulted in altered expression of autophagy genes and accumulation of mitochondrial DNA [66]. ZMIZ1 was also recently shown to inhibit autophagy in cultured neurons via sumoylation of NLRP3 [67]. However, the biological significance of ZMIZ1 in brain mitophagy, its molecular contribution to the synergy between LB and NFT pathology, and its potential context-dependent protective role against mitophagy dysfunction remain largely unexplored and warrant future investigation.

Although we reveal a synergistic interplay between genetic factors and comorbid pathologies that drives regional vulnerability to mitophagy alterations in the autopsy-defined LBD brain, our study also has certain limitations. Further analysis using complementary, independent methods beyond the primary immunohistochemical readout would strengthen and expand the current findings. By characterizing pS65-Ub labeled mitochondrial damage in postmortem brain, we provide a snapshot of the cumulative burden on PINK1–PRKN-directed mitochondrial quality control at the end-stage of disease. Spatial multiomics analysis at single-cell resolution will help better define pS65-Ub substrates and resolve the complexity of these interactions at the subcellular level. To dissect the temporal and mechanistic contributions to the synergy between genetic variants and neuropathologies, dynamic, real-time analyses in model systems are needed for precise monitoring of mitochondrial damage, pS65-Ub production, trafficking of labeled cargo, and autophagic degradation over the course of disease progression. Our current analysis was also limited to mature inclusions (LBs, SPs, and NFTs), while emerging evidence suggests that oligomeric species or fibrils may exert greater cytotoxicity than mature inclusions [68–71]. Future studies on these pathological species will provide more insight into the pathological impact on mitophagy and define potential pathological, non-degradable pS65-Ub substrates. Other comorbid pathologies such as TDP-43 and cerebral amyloid angiopathy, both implicated in different aspects of mitochondrial function [72–75], may also be considered in future studies. While pS65-Ub is an intriguing aging and disease marker, much remains to be resolved to elucidate the nature and origin of the pS65-Ub signal and to refine our interpretation of its accumulation. Nevertheless, given the clinical, genetic, and pathological overlap among PD, LBD, and AD, targeting mitochondrial health and mitophagy may offer a promising common therapeutic strategy. A better understanding of the molecular architecture of pathological aggregates and their impact on mitochondrial homeostasis could refine our approaches to develop disease-modifying therapies.

## Supplementary Information

Below is the link to the electronic supplementary material.

Supplementary file1 (PDF 679 KB)

## Data Availability

The datasets used and/or analyzed during the current study are available from the corresponding author Dr. Wolfdieter Springer ([Springer.Wolfdieter@mayo.edu] (mailto: Springer.Wolfdieter@mayo.edu)) upon request.

## Abbreviations

AD: Alzheimer’s disease
LBD: Lewy body dementia
LB: Lewy body
NFT: Neurofibrillary tangle
PD: Parkinson’s disease
pS65-Ub: Phosphorylated ubiquitin at serine 65
SP: Senile plaque
