# Supplementary material for: Genetic factors and comorbid pathologies interact to drive regional mitophagy alterations in Lewy body dementia
Source: Acta Neuropathol. 2025 Dec 1;150(1):59. doi: 10.1007/s00401-025-02964-6 (PMC12669339; doi:10.1007/s00401-025-02964-6)
Supplement: Supplementary file 1 — Supplementary file1 (PDF 679 KB) [file 401_2025_2964_MOESM1_ESM.pdf]

Supplemental Table 1 Associations of pS65-Ub with neuropathology in non-carriers and carriers of *APOE4*

Association with pS65-Ub when adjusting for age at death, sex, and other neuropathology[1]	Non-carriers of *APOE*4 (N=186)		Carriers of *APOE4* (N=166)		Interaction p-value between the variable of interest and *APOE4*
	β (95% CI)	p-value	β (95% CI)	p-value	
**Hippocampus**					
LB density	0.10 (-0.02, 0.21)	0.10	0.18 (0.04, 0.33)	**0.015**	0.071
SP density	0.16 (0.04, 0.29)	**0.013**	0.28 (0.09, 0.48)	**0.0047**	0.019
NFT density	0.38 (0.25, 0.51)	**1.16×10^{-7}**	0.66 (0.43, 0.89)	**1.10×10^{-7}**	**0.0078**
**Amygdala**					
LB density	0.14 (0.04, 0.24)	**0.0051**	0.17 (0.00, 0.35)	0.048	0.70
SP density	0.24 (0.11, 0.38)	**0.0005**	0.32 (0.15, 0.49)	**0.0002**	0.53
NFT density	0.32 (0.19, 0.44)	**1.45×10^{-6}**	0.24 (0.05, 0.42)	**0.013**	0.98

β - regression coefficient; CI - confidence interval; LB - Lewy body; SP - senile plaque; NFT - neurofibrillary tangle. β values, 95% CIs, and p-values result from linear regression models. β coefficients are interpreted as the increase in mean pS65-Ub level (on the cube root scale and after subsequent scaling to mean=0 and standard deviation=1) corresponding to each 1-standard deviation increase (after cube root transformation when applicable) of LB density, SP density, or NFT density.

[1] Adjustment for other neuropathology was done as follows. Models directly involving LB density were adjusted for SP and NFT density; models directly involving SP density were adjusted for LB and NFT density; models directly involving NFT density were adjusted for LB and SP density. When assessing the interaction between presence of the *APOE4* allele and the given variable of interest (LB, SP, or NFT density) with regard to association with pS65-Ub level, the aforementioned linear regression models were utilized in the entire series of LBD cases, with additional inclusion of covariates for presence of the *APOE4* allele and the interaction between presence of the *APOE4* allele and the given variable of interest. For tests of association, p-values ≤0.0167 were considered as statistically significant after applying a Bonferroni correction for multiple testing for the three measures (LB, SP, and NFT density) that were examined for association with pS65-Ub level in a given brain region for the separate groups of non-carriers and carriers of *APOE4*. For tests of interaction, p-values ≤0.0167 were considered as statistically significant after applying a Bonferroni correction for multiple testing for the three tests of interaction that were performed in a given brain region. Significant p-values are shown in bold.

Supplemental Table 2 Associations of pS65-Ub with neuropathology in non-carriers and carriers of *ZMIZ1* rs6480922 minor allele

Association with pS65-Ub when adjusting for age at death, sex, and other neuropathology[1]	Non-carriers of *ZMIZ1* rs6480922 minor allele (N=206)		Carriers of *ZMIZ1* rs6480922 minor allele (N=148)		Interaction p-value between the variable of interest and *ZMIZ1* rs6480922
	β (95% CI)	p-value	β (95% CI)	p-value	
**Hippocampus**					
LB density	0.13 (-0.00, 0.25)	0.055	0.17 (0.03, 0.31)	**0.016**	0.59
SP density	0.21 (0.06, 0.36)	**0.0049**	0.21 (0.04, 0.37)	**0.014**	0.58
NFT density	0.47 (0.31, 0.63)	**2.70×10^{-8}**	0.51 (0.32, 0.71)	**6.66×10^{-7}**	0.31
**Amygdala**					
LB density	0.14 (0.01, 0.26)	0.029	0.17 (0.03, 0.32)	0.017	0.90
SP density	0.34 (0.20, 0.49)	**6.10×10^{-6}**	0.27 (0.11, 0.43)	**0.0014**	0.62
NFT density	0.29 (0.15, 0.43)	**0.0001**	0.34 (0.17, 0.51)	**0.0001**	0.98

β - regression coefficient; CI - confidence interval; LB - Lewy body; SP - senile plaque; NFT - neurofibrillary tangle. β values, 95% CIs, and p-values result from linear regression models. β coefficients are interpreted as the increase in mean pS65-Ub level (on the cube root scale and after subsequent scaling to mean=0 and standard deviation=1) corresponding to each 1-standard deviation increase (after cube root transformation when applicable) of LB density, SP density, or NFT density.

[1] Adjustment for other neuropathology was done as follows. Models directly involving LB density were adjusted for SP and NFT density; models directly involving SP density were adjusted for LB and NFT density; models directly involving NFT density were adjusted for LB and SP density. When assessing the interaction between presence of the *ZMIZ1* rs6489022 minor allele and the given variable of interest (LB, SP, or NFT density) with regard to association with pS65-Ub level, the aforementioned linear regression models were utilized in the entire series of LBD cases, with additional inclusion of covariates for presence of the *ZMIZ1* rs6480922 minor allele and the interaction between presence of the *ZMIZ1* rs6480922 minor allele and the given variable of interest. For tests of association, p-values ≤0.0167 were considered as statistically significant after applying a Bonferroni correction for multiple testing for the three measures (LB, SP, and NFT density) that were examined for association with pS65-Ub level in a given brain region for the separate groups of non-carriers and carriers of the minor allele of *ZMIZ1* rs6480922. For tests of interaction, p-values ≤0.0167 were considered as statistically significant after applying a Bonferroni correction for multiple testing for the three tests of interaction that were performed in a given brain region. Significant p-values are shown in bold.

Supplemental Table 3 Interactions of α-synuclein with amyloid and tau pathology for associations with pS65-Ub in Lewy body dementia

SP or NFT density subgroup (Low: ≤median, High: >median)		Association between LB density and pS65-Ub		Interaction p-value
		β (95% CI)	p-value	
**Amygdala**				
SP density	Low (N=188)	0.12 (0.04, 0.21)	0.0052	0.10
	High (N=183)	0.20 (0.01, 0.39)	0.035	
NFT density	Low (N=192)	0.07 (-0.00, 0.15)	0.063	**0.025**
	High (N=179)	0.25 (0.04, 0.47)	0.021	
**Hippocampus**				
SP density	Low (N=140)	0.09 (-0.01, 0.19)	0.063	0.054
	High (N=135)	0.15 (0.00, 0.30)	0.048	
NFT density	Low (N=156)	0.15 (0.07, 0.24)	0.0006	0.92
	High (N=119)	0.07 (-0.09, 0.22)	0.40	

β - regression coefficient; CI - confidence interval; LB - Lewy body; SP - senile plaque; NFT - neurofibrillary tangle. β values, 95% CIs, and p-values result from linear regression models that were adjusted for age at death, sex, SP density, and NFT density. β coefficients are interpreted as the increase in mean pS65-Ub level (on the cube root scale and after subsequent scaling to mean=0 and standard deviation=1) corresponding to each 1-standard deviation increase (after cube root transformation when applicable) of LB density. When assessing the interaction between LB and SP density with regard to association with pS65-Ub level, the aforementioned linear regression models were utilized in the entire series of LBD cases, with additional inclusion of a covariate for the interaction between LB and SP density. When assessing the interaction between LB and NFT density with regard to association with pS65-Ub level, the aforementioned linear regression models were utilized in the entire series of LBD cases, with additional inclusion of a covariate for the interaction between LB and NFT density. P-values ≤0.025 were considered as statistically significant after applying a Bonferroni correction for multiple testing for the two tests of interaction that were performed in a given brain region. Significant p-values are shown in bold.

Supplemental Table 4 Interactions of α-synuclein and tau pathology for associations with pS65-Ub in the amygdala in different genetic groups

Genetic status	NFT density subgroup (Low: ≤median, High: >median)	Association between LB density and pS65-Ub		Interaction p-value	Test of three-way interaction
		β (95% CI)	p-value		
**Minor allele of *ZMIZ1* rs6480922**					
Non-carriers	Low (N=106)	0.10 (0.01, 0.20)	0.039	0.65	**0.0039**
	High (N=100)	0.03 (-0.29, 0.35)	0.85		
Carriers	Low (N=79)	0.02 (-0.12, 0.15)	0.81	**0.0005**	
	High (N=69)	0.48 (0.17, 0.80)	0.0030		
***APOE4* allele**					
Non-carriers	Low (N=120)	0.10 (0.01, 0.19)	0.031	0.31	0.54
	High (N=66)	0.14 (-0.12, 0.41)	0.28		
Carriers	Low (N=65)	0.03 (-0.13, 0.20)	0.69	0.18	
	High (N=101)	0.26 (-0.10, 0.62)	0.15		

β - regression coefficient; CI - confidence interval; LB - Lewy body; NFT - neurofibrillary tangle. NFT density was dichotomized using the sample median. β values, 95% CIs, and p-values result from linear regression models that were adjusted for age at death, sex, SP density, and NFT density. β coefficients are interpreted as the increase in mean pS65-Ub level (on the cube root scale and after subsequent scaling to mean=0 and standard deviation=1) corresponding to each 1-standard deviation increase (after cube root transformation when applicable) of LB density. When assessing the interaction between LB and NFT density with regard to association with pS65-Ub level in the separate groups of carriers and non-carriers of the minor allele of *ZMIZ1* rs6480922 or *APOE4*, the aforementioned linear regression models were utilized in the entire series of LBD cases, with additional inclusion of a covariate for the interaction between LB and NFT density. When evaluating whether these interactions were consistent between carriers and non-carriers of the minor allele of *ZMIZ1* rs6480922 or *APOE4*, we added an interaction with presence of the minor allele of *ZMIZ1* rs6480922 or *APOE4* into the interaction terms of the aforementioned regression models (i.e., a three-way interaction). P-values ≤0.025 were considered as statistically significant after applying a Bonferroni correction for multiple testing for the two three-way interactions that were assessed. Significant p-values are shown in bold.

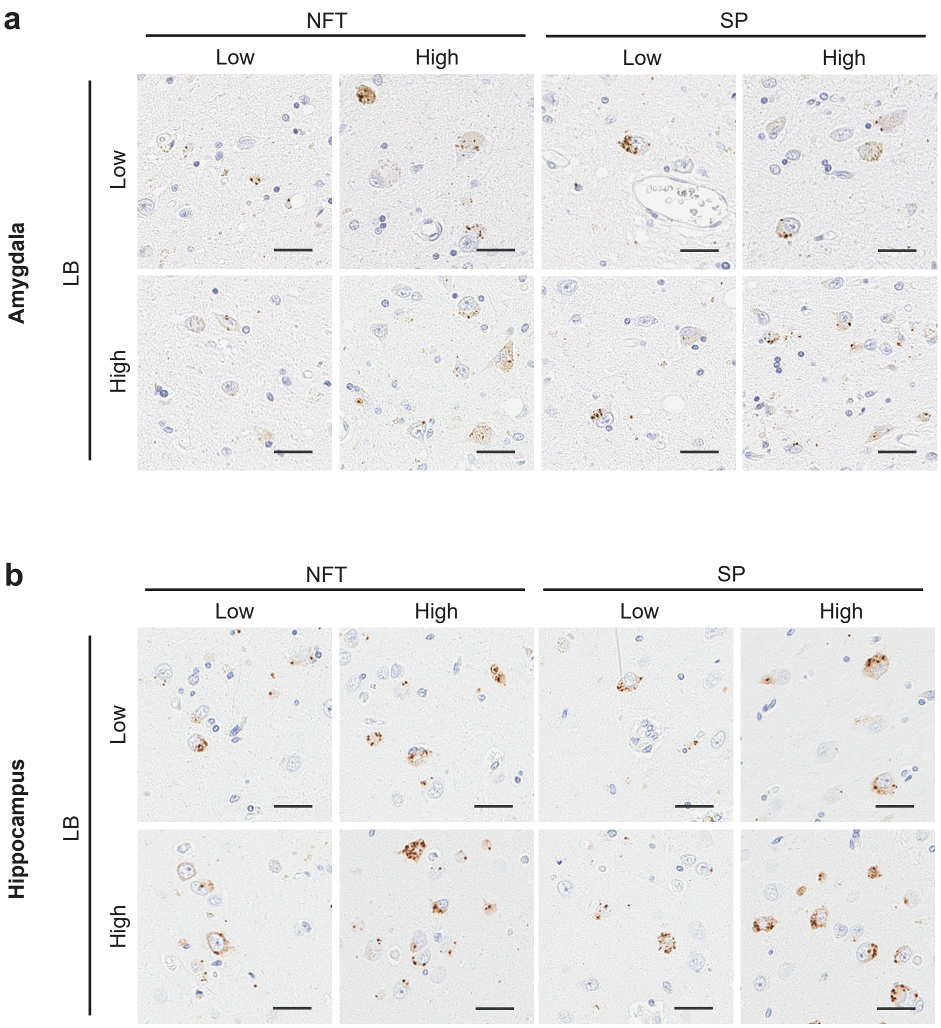

Supplemental Fig. 1 Interaction of comorbid neuropathologies on pS65-Ub accumulation in DLB. Representative images of pS65-Ub staining in (**a**) the amygdala and (**b**) hippocampus with low or high LB, NFT, or SP densities. Scale bar: 30 μm
